# Can cholera ‘hotspots’ be converted to cholera ‘coldspots’ in cholera endemic countries? The Matlab, Bangladesh experience

**DOI:** 10.1016/j.ijid.2020.02.055

**Published:** 2020-06

**Authors:** K. Zaman, Deok Ryun Kim, Mohammad Ali, Faisal Ahmmed, Justin Im, Md Taufiqul Islam, Ashraful Islam Khan, Md Yunus, Md Alfazal Khan, Florian Marks, Firdausi Qadri, Jerome Kim, John D. Clemens

**Affiliations:** a International Centre for Diarrhoeal Disease Research, Bangladesh, Dhaka, Bangladesh; b International Vaccine Institute, Seoul, Republic of Korea; c Johns Hopkins University, Baltimore, MD, USA; d Department of Medicine, University of Cambridge, Cambridge, UK; e UCLA Fielding School of Public Health, Los Angeles, CA 90095-1772, USA; f Korea University College of Medicine, Seoul 02841, South Korea

**Keywords:** Cholera, Lantrices, Environmental factors, Observation, Augmented, Incidence

## Abstract

**Background:**

Cholera remains a major public health threat in low- and middle-income countries. The World Health Organization (WHO) has recently launched a global initiative to end preventable cholera by 2030. Key to the success of this initiative will be the elimination of cholera transmission in cholera ‘hotspots’ with regularly recurrent disease; this can be achieved via improved surveillance to define hotspot populations, through the use of oral cholera vaccines, and through the implementation of improved water, sanitation, and hygiene (WASH).

**Methods:**

This study was performed to analyze the trend in cholera incidence during the years 1974–2018 in Matlab, Bangladesh (defined population of about 200 000) that has been recognized as one of the world’s cholera hotspots. During this period, Matlab has maintained a demographically defined population and comprehensive, culture-based surveillance for cholera, supplemented by periodic surveys to characterize the socioeconomic status of the population, as well as water sources and facilities for defecation.

**Results:**

Cholera transmission has nearly been eliminated in Matlab, despite a continuing high cholera burden in many other parts of Bangladesh and despite trends of increasing ambient and sea surface temperatures, which are known to increase cholera incidence. Concomitantly, the socioeconomic status of the population has increased modestly, and the use of simple tubewells for drinking water has reached 95% and the installation of sanitary latrines has reached 85%.

**Conclusions:**

The factors responsible for the decline in cholera are difficult to pinpoint precisely, but this decline has occurred with the installation of inexpensive improvements in water sources and latrines and despite environmental factors that should have augmented cholera incidence. These observations lend optimism to the current global initiative to end preventable cholera by 2030.

## Highlights

• Matlab, Bangladesh was a well-documented cholera ‘hotspot’ – an area of regularly recurrent cholera with a high annual incidence – for many decades.
• In recent years, cholera has declined to negligible levels in Matlab, despite persistently high rates in many areas of Bangladesh and despite increasing local ambient and sea surface temperatures, which favor a high cholera incidence.
• The decline occurred following the provision of low-cost tubewells for the supply of water and inexpensive sanitary latrines to a high proportion of the population.
• These observations lend optimism to the success of the World Health Organization current global initiative to end preventable cholera by 2030.

Cholera is an acute diarrheal illness, transmitted by fecal–oral route and caused by *Vibrio cholerae* (mostly *V. cholerae* O1). Cholera remains a major public health threat in low- and middle-income countries (LMICs), causing approximately 100 000 deaths annually (Ali et al., 2015). The global caseload and death toll from cholera have shown no signs of decline in the past two decades (Lonappan et al., 2019). It is axiomatic that the provision of clean water and adequate sanitation, and the maintenance of good personal and food hygiene, will halt the transmission of cholera. Indeed, affluent countries of the world have succeeded in doing so, primarily through the installation of municipal systems to provide clean water and to collect and treat sewerage. The situation is quite different in LMICs, despite several global initiatives to improve access to clean water and adequate sanitation. Worldwide, it is estimated that 844 million people still lack access to safe drinking water sources, more than two billion are drinking water from sources that are fecally contaminated, and 2.4 billion people have no basic sanitation facilities (WHO/UNICEF, 2017).

Recently, a global stockpile of killed oral cholera vaccines (OCVs) has been created to aid in the effort to control cholera. Since 2013, over 50 million doses of these vaccines have been deployed for the control of both endemic and epidemic cholera and in humanitarian emergencies in Asia, Africa, the Middle East, and Haiti. When evaluated in these settings, the vaccines have consistently been shown to be effective (Bhattacharya et al., 2013, Azman et al., 2016, Qadri et al., 2015, Sévère et al., 2016). Yet, it is recognized that OCVs are a near-term approach to controlling cholera, and the number of doses in the stockpile is sufficient only for a very small fraction of the populations at risk of cholera.

Against this backdrop, the World Health Organization (WHO) has proposed a bold initiative to end cholera by 2030 (https://www.who.int/cholera/publications/global-roadmap/en/ (accessed November 12, 2019). Key to this initiative is the assumption that cholera in endemic countries is driven by ‘hotspots’ (i.e., subpopulations exhibiting recurrent cholera, year after year), that these hotspots can be identified through improved cholera surveillance, and that the use of OCVs and implementation of improved water, sanitation, and hygiene (WASH) in the hotspot populations can prevent cholera transmission, benefitting not only the hotspot populations, but also populations residing outside the hotspots to which cholera may spread. As the widespread installation of municipal water and sewerage systems in LMICs is too expensive for contemplation before 2030, it might be questioned whether cholera transmission in cholera hotspots in endemic countries can be controlled with more affordable WASH investments. Herein we describe the example of Matlab, Bangladesh, a well-documented cholera hotspot for decades, from which cholera has nearly disappeared in recent years, in the context of a country that overall remains highly endemic for cholera.

Bangladesh is one of the most cholera-endemic countries in the world. The Ganges delta, which India and Bangladesh straddle, is known to be the homeland of cholera. Six of the seven pandemics since the 19th century have originated here. According to the WHO, the entire population of Bangladesh (approximately 160 million people) is at risk of frequent flooding, a common antecedent of cholera outbreaks, and it has been estimated that there are about 3300 cholera deaths annually (Ali et al., 2015, Khan et al., 2018). The International Centre for Diarrhoeal Disease Research, Bangladesh (icddr,b) operates two hospitals in Dhaka City in which 15 000–20 000 cholera cases are currently seen annually (icddr,b Dhaka Hospital surveillance data, 2018). Given the generally inadequate water quality, hygiene, and sanitation for the country’s most impoverished populations, who bear the brunt of this burden, in addition to the effects of global warming, particularly pronounced for Bangladesh and related to an increased incidence of cholera, this is perhaps not surprising.

**Figure 1:**
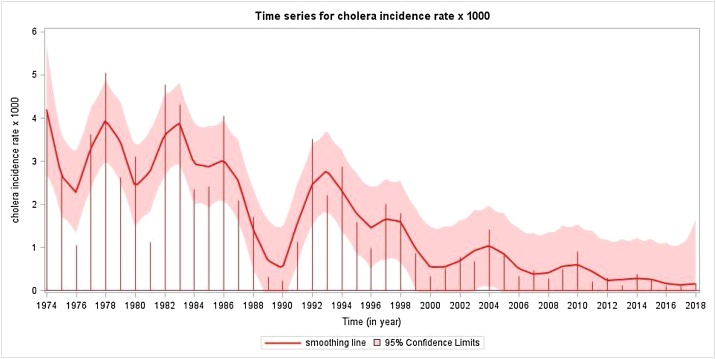
Smoothed yearly incidence of cholera at Matlab, 1974–2018. A linear regression model was used. For the overall period, the slope (−0.0827 (SE 0.0108), *t*-value = −7.75, *p* < 0.001) of time (year) decreased significantly. From 1974 to 1993, the slope (−0.0968 (SE 0.0525), *t*-value = −1.84, *p* = 0.0820) of time (year) showed a marginally significant decrease. From 1994 onward, the slope (−0.0780 (SE 0.0148, *t*-value = −5.26, *p* < 0.001) of time (year) decreased significantly.

**Figure 2:**
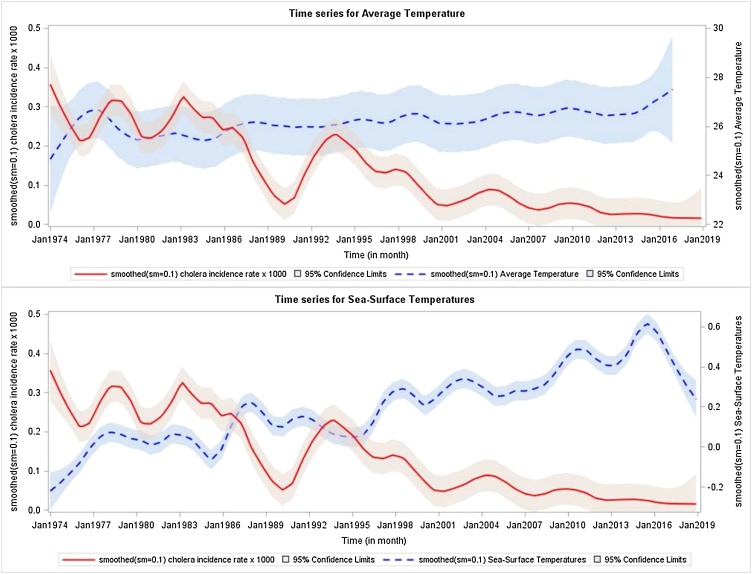
Smoothed monthly incidence of cholera and the average temperature at Matlab and the sea surface temperature of the Indian Ocean, 1974–2018.

What is surprising is the secular trend of cholera in Matlab, a rural field study area of the icddr,b with a population of about 200 000 persons (currently 239 000), located 30 miles southeast of Dhaka. Since the 1960s, Matlab has been one of the world’s ‘go-to’ sites for evaluating cholera vaccines, because of its consistently high incidence of cholera (Glass et al., 1982). Indeed, from the 1960s to the 1980s, six large-scale cholera vaccine efficacy trials were conducted by the icddr,b at this site (Zaman and Md, 1995). Although all of these trials enrolled substantial fractions of the Matlab population, none of them tested vaccines whose efficacy persisted beyond 3 years. Nonetheless, during the past three decades, Matlab, which has maintained ongoing comprehensive surveillance for cholera since its inception, has seen a dramatic decline in cholera incidence (Figure 1). Prior to the 1990s, although cholera incidence fluctuated year by year, the annual incidence of treated cholera was typically at least 1 case per 1000, and often several cases per 1000. The last cholera vaccine trial to be conducted in Matlab was initiated in 1985 (Clemens et al., 1986). Although the OCVs tested were safe and effective, protection did not persist beyond 3 years after dosing, in 1988. The trend of decline in incidence since 1994 is highly significant (slope −0.0780, *p* < 0.001 by linear regression). At the same time, there has been evidence of rising ambient temperatures in Matlab, as well as rising sea surface temperatures in the Indian Ocean, on which Bangladesh sits, both previously associated with higher rates of cholera in Matlab (Figure 2).

**Figure 3:**
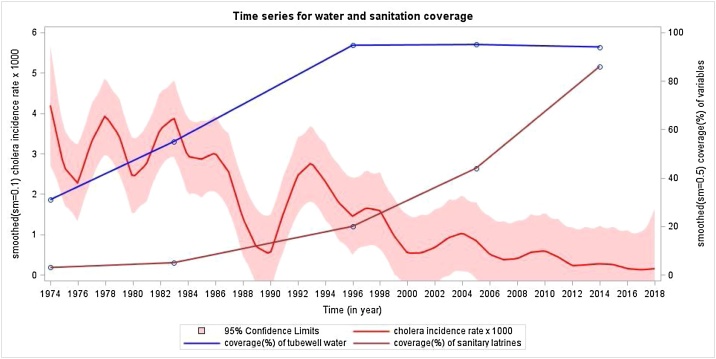
Smoothed yearly incidence of cholera and the household level coverage with tubewell water and sanitary latrines in Matlab, Bangladesh, 1974–2018.

**Figure 4:**
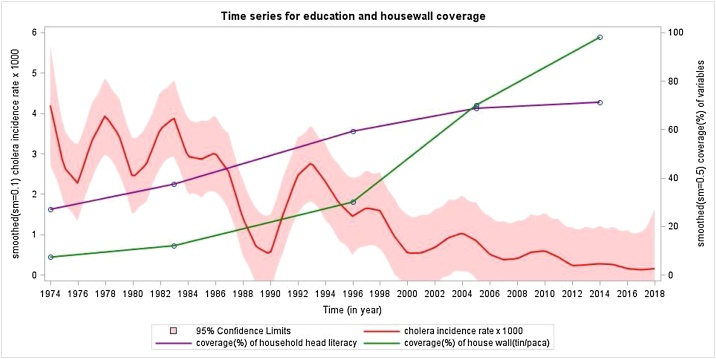
Smoothed yearly incidence of cholera and the household head literacy and house wall structure in Matlab, Bangladesh, 1974–2016.

What countervailing factors since the 1990s might have accounted for this decline in cholera? Since our surveillance captures only cholera severe enough to prompt patients to seek care, it is possible that the use of oral rehydration solution (ORS) at home could have mitigated the illness and averted seeking of treatment. Matlab was the site for initial demonstration of the efficacy of ORS for cholera in clinical settings in the 1970s, and the use of ORS in homes has been encouraged since 1980. However, coverage rates of ORS at home for childhood diarrhea in Matlab were only in the range of 40% to 60% in surveys done between 1998 and 2016. Furthermore, ORS is thought to be less commonly used in adults than in children. Another possibility might be the increasing prevalence of salutary WASH facilities and practices. Although data on secular trends in hygiene are not available for Matlab, there have been improvements in the installation of tubewells for clean water and sanitary hygiene (Figure 3). Notably, these improvements have been very inexpensive relative to the costs of installing modern municipal water and sanitation systems. Finally, the socioeconomic status of the Matlab has improved over time, as reflected by improving levels of education and construction of homes (usually small and modest) with more expensive concrete materials (Figure 4). That said, those changes are visible but rather modest.

Although it is not possible to pinpoint precisely which factors have been directly responsible for the marked temporal decline in cholera over the years in Matlab, it is striking that this decline occurred in the face of environmental and climatic factors that should have augmented cholera incidence, and in a country in which endemic cholera continues to be a major public health problem. Improvements in WASH undoubtedly have played an important role, but it is noteworthy that the decline occurred without the benefit of using effective OCVs and with relatively modest investments in WASH. Likely, improvements in socioeconomic status and education have also played a supportive role, as has awareness about diarrhea prevention in a site that has supported diarrhea studies for years. Further research is needed to study in greater detail the factors responsible for this remarkable decline, including behaviors responsible for preventing cholera and mitigating its severity. In aggregate, these observations lend optimism to the argument that radical investments in infrastructure and socioeconomic development are not needed to eliminate cholera in endemic hotspots, and predict that the current WHO goal to eliminate cholera by 2030 using a combination of improved surveillance, augmented WASH, and use of OCVs may well be achievable if affected countries fully engage in these cholera control activities.

Author contributions: The International Centre for Diarrhoeal Disease Research (icddr,b) established a collaboration with Deok Ryun Kim (DRK), Justin Im (JI), Florian Marks (FM), and Jerome Kim (JK) from the International Vaccine Institute (IVI); the IVI and icddr,b engaged in extensive discussions, resulting in the idea for the paper and the strategy to write it. All of the authors reviewed the paper. DRK, Mohammad Ali (MA), and Faisal Ahmed (FA) contributed to the analysis of the data, and reviewed and approved the manuscript. Md Taufiqul Islam (MTI) and Ashraful Islam Khan (AIK) reviewed and approved the manuscript. Md Yunus (MY) worked at Matlab for 40 years and headed Matlab; he was actively involved with the hospital surveillance data. He provided surveillance data, and reviewed and approved the manuscript. Md Alfazal Khan (MAK) also worked at Matlab hospital for 20 years and is the current head of Matlab. He also provided surveillance data, and reviewed and approved the manuscript. Firdausi Qadri (FQ) is a cholera expert and provided input in the writing, review, and approval of the manuscript. K. Zaman (KZ) worked many years at Matlab, coordinated the gathering of the data, and was involved in the writing, review, and approval of the manuscript. John D. Clemens (JCD) led the team, provided the idea for the paper, and wrote, reviewed, and gave final approval of the manuscript.

## Declarations

*Funding:* The research was supported by the Diseases of the Most Impoverished Program of the Bill & Melinda Gates Foundation. This study was supported by a grant (OPP1171432) from the Bill & Melinda Gates Foundation.

*Conflict of interest:*The authors state no commercial or other association that might pose a conflict of interest with regard to the findings presented in this manuscript.
